# Flavonoids, Potential Bioactive Compounds, and Non-Shivering Thermogenesis

**DOI:** 10.3390/nu10091168

**Published:** 2018-08-25

**Authors:** Hye Won Kang, Sang Gil Lee, Dammah Otieno, Kyoungsoo Ha

**Affiliations:** 1 Food and Nutritional Sciences, Department of Family and Consumer Sciences, North Carolina Agricultural and Technical State University, Greensboro, NC 27411, USA; kha@ncat.edu; 2 Department of Food Science and Nutrition, Pukyong National University, Busan, 48513, Korea; sglee1125@pknu.ac.kr; 3 Department of Applied Science and Technology, North Carolina Agricultural and Technical State University, Greensboro, NC 27411, USA; dotieno@aggies.ncat.edu

**Keywords:** non-shivering thermogenesis, brown adipose tissue, beige adipocytes, obesity, flavonoids

## Abstract

Obesity results from the body having either high energy intake or low energy expenditure. Based on this energy equation, scientists have focused on increasing energy expenditure to prevent abnormal fat accumulation. Activating the human thermogenic system that regulates body temperature, particularly non-shivering thermogenesis in either brown or white adipose tissue, has been suggested as a promising solution to increase energy expenditure. Together with the increasing interest in understanding the mechanism by which plant-derived dietary compounds prevent obesity, flavonoids were recently shown to have the potential to regulate non-shivering thermogenesis. In this article, we review the latest research on flavonoid derivatives that increase energy expenditure through non-shivering thermogenesis.

## 1. Introduction

Obesity is a growing health problem worldwide [1]. Excessive body fat, which is referred to as obesity, impairs normal metabolic regulation, which increases risk factors for the development of other metabolic disorders, such as type 2 diabetes, insulin resistance, atherosclerosis, and cardiovascular diseases [1]. Given its seriousness as a health issue, various strategies have attempted to prevent and reduce obesity such as diet programs, surgery, and medications. However, these current strategies have limitations such as side effects, requirement of long-term efforts, and appropriateness for certain age ranges. Consequently, there remains a need to discover new methods to control obesity. One new way to regulate fat accumulation is to activate the non-shivering thermogenesis function of brown adipose tissue (BAT). Non-shivering thermogenesis is a cold-induced heat generating process that was initially found in newborn infants and hibernating animals against hypothermia [2,3]. Due to the re-discovery of metabolically active BAT in adult humans, which burns stored fat and diet-driven circulating fat, BAT has been re-evaluated as a potential tissue for the prevention and improvement of metabolic diseases, such as obesity, diabetes, and cardiovascular diseases [4,5,6,7].

Brown adipocytes are derived from the same precursor cells, which express myogenic factor 5 (*Myf5*), as muscle cells, which are interchangeable with skeletal myoblasts via positive regulatory domain containing 16 (PRDM16), a transcriptional factor [8,9]. However, the mitochondria of brown adipocytes are distinct from those of other oxidative tissues; for example, they differ from those of muscle tissues due to their expression of uncoupling protein 1 (UCP1), a BAT-specific protein in the mitochondrial inner-membrane. Mitochondria generally produce ATP by ATP synthase, which phosphorylates ADP when electron transport generates a proton gradient across the inner-membrane of mitochondria. UCP1 allows the mitochondria to dissipate heat by diminishing the proton gradient, which uncouples substrate oxidation in oxidative phosphorylation [8,9]. Unlike UCP1, which is dominantly expressed in BAT, its homologues, UCP2 and UCP3, are also expressed in the stomach, spleen, pancreas, and lung and in skeletal muscle, respectively. The physiological functions of UCP2 and UCP3 include reducing production of reactive oxygen species and decreasing insulin secretion in β-cells of the pancreas by increasing proton conductance in the mitochondrial inner-membrane [10]. Although UCP2 and UCP3 expression are increased by fatty acids, which indicates a possible relationship with energy metabolism, there is no clear understanding of whether UCP2 and UCP3 have thermogenic functions.

The development of brown adipocytes is facilitated by various transcriptional factors and proteins such as bone morphogenetic protein (BMP) 7, PRDM16, peroxisome proliferator-activated receptor gamma (PPARγ) coactivator 1 alpha (PGC1α), cell death-inducing DNA fragmentation factor alpha (DFFA)-like effector a (CIDEA), and PPARα [11]. With the activation of BMP7, brown adipocytes are developed from multipotent mesenchymal cells and then differentiated into cells that have the identity of brown adipocytes by PRDM16. PRDM16 is induced by PPARγ, a necessary transcriptional factor during white adipogenesis that is activated by CCAAT/enhancer-binding protein beta (C/EBPβ), in cooperation with C/EBPα and PPARα [11,12]. In the differentiation process of brown adipocytes, PGC1α, the thermogenic regulator is increased, which activates UCP1 and mitochondrial biogenesis [11]. PGC1α may be induced by PPARα, a transcriptional factor that regulates lipolysis and fatty acid oxidation [12]. PGC1α was also induced by increased circulating levels of fibroblast growth factor (FGF) 21, which leads to an increase in non-shivering thermogenesis [13]. Along with the differentiation process and substrate oxidation, CIDEA plays a role in developing multilocular lipid droplets in BAT, which are a characteristic of brown adipocytes [14,15,16]. Unlike brown adipocytes in BAT, some *Myf5*-negative adipocytes in white adipose tissue (WAT) have also shown BAT phenotypes in response to certain stimuli, e.g., cold; these are called beige adipocytes [17]. Similar to its role in BAT, PRDM16 modulates white adipocytes during adipogenesis in the cooperative system with PPARγ, C/EBPβ, and C/EBPα to have the characteristics of brown adipocytes by acquiring BAT-selective proteins, e.g., PRDM16, UCP1, PGC1α, and CIDEA, which allows the cells to display non-shivering thermogenesis activity [17]. PRDM16 is also induced by BMP7 in white progenitor cells [18]. Beyond brown-selective proteins, beige adipocytes show the increased expression of some proteins such as T-box transcription factor 1 (TBX) 1, transmembrane protein (TMEM) 26, Cbp/p300-interacting transactivator (CITED) 1, and CD137/tumor necrosis factor receptor superfamily member 9 (TNFRSF9), which are indicated as beige-selective proteins [19].

When the body is exposed to cold temperature, the hypothalamus in the sympathetic nervous system of the brain releases norepinephrine, which binds the β_3_-adrenergic receptor (AR) to activate cyclic AMP (cAMP). This subsequently activates protein kinase C (PKC) and PKA by phosphorylation, which increases the phosphorylation of hormone sensitive lipase (HSL) that breaks down triacylglycerols (TAGs) into glycerol and free fatty acids [20]. The free fatty acids directly activate UCP1 expression or substrate oxidation to increase the level of nicotinamide adenine dinucleotide (NADH), which eventually produce heat by UCP1-mediated uncoupled oxidative phosphorylation, called non-shivering thermogenesis [20]. Because the non-shivering thermogenesis pathway is tightly correlated with fatty acid utilization, it is also regulated by other hormones and regulators that play roles in energy metabolism, such as thyroid hormone and AMP-activated protein kinase (AMPK). Thyroid hormone plays a role in the regulation of energy metabolism, which showed a possible correlation with the regulation of non-shivering thermogenesis on BAT and the browning of WAT [21]. Thyroid hormone increases UCP1 by regulating thyroid hormone receptor or deiodinase 2 (DIO2) [22]. Hyperthyroid mice showed increased BAT activity, but this change was not found in hypothyroid mice. WAT browning was shown in both hyper- and hypothyroid mice [21]. AMPK is the main regulator in energy balance, which is activated by low energy levels (high AMP/ATP ratio) [23]. Activated AMPK, particularly by phosphorylation, inhibits acetyl-coenzyme A carboxylase (ACC) by phosphorylation, which prevents fatty acid synthesis and increases fatty acid oxidation and mitochondrial biogenesis, respectively [23]. Silent mating type information regulation 2 homolog (SIRT) 1, which is activated by a high NAD^+^/NADH ratio activates PGC1α by deacetylation, which continually activates UCP1-mediated non-shivering thermogenesis [23]. Non-shivering thermogenesis is positively associated with lipolysis and fatty acid oxidation, which switches the body’s metabolism to efficiently burn fat as heat [3]. Therefore, pharmaceutical studies have focused on developing drugs to activate non-shivering thermogenesis in BAT and/or WAT that improve metabolic disorders. Although cold and exercise are well-known stimuli that increase non-shivering thermogenesis, phytochemicals, such as resveratrol and curcumin have shown the potential to activate BAT and to exert a browning effect in WAT [24,25,26].

**Figure 1:**
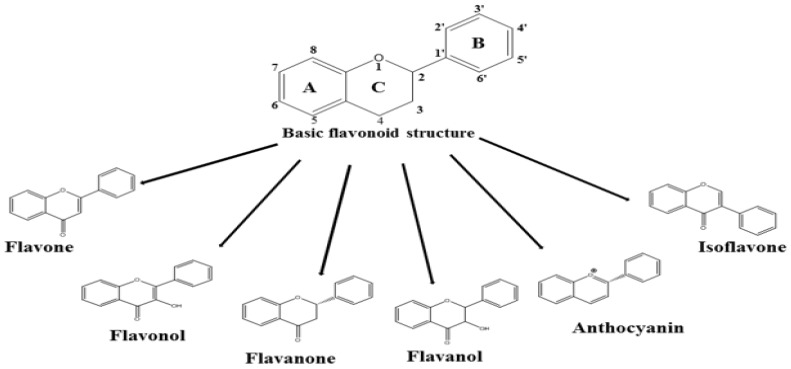
Structure of Flavonoids.

Of the known dietary compounds, flavonoids are the most popular phytochemicals found abundantly in fruits and vegetables. The general structure of flavonoids is C6-C3-C6, which consists of two phenyl rings (A and B) and one heterocyclic ring (C) (Figure 1) [27]. Based on their substitution pattern, flavonoids are classified into six categories: flavones (e.g., luteolin and apigenin), flavonols (e.g., quercetin and kaempferol), flavanones (e.g., naringenin and hesperidin), flavanols (e.g., catechin and epicatechin), anthocyanins (e.g., cyanidin and delphinidin), and isoflavones (e.g., genistein and daidzein) [27]. Although each individual flavonoid has specific health benefits; many phytochemicals that are classified as flavonoids exert an anti-obesity effect by regulating the oxidation, synthesis, uptake, and transport of fatty acids [27]. Individuals with a higher flavonoid intake show lower obesity indicators, e.g., a lower body mass index, and decreased levels of C-reactive protein, a marker of inflammation [28]. Because no previous review article specifically focused on the effects of flavonoids on BAT activation and adipocyte browning, this article reviews all subgroups of flavonoids that have shown the potential to increase energy expenditure by increasing non-shivering thermogenesis.

## 2. Effects of Flavonoids on Non-Shivering Thermogenesis

### 2.1. Flavones

#### 2.1.1. Primary Food Sources and Daily Intake

The structure of the flavone backbone is a 2-phenylchromen-4-one (2-phenyl-1-benzopyran-4-one). Flavones are classified into various subgroups based on the side chains attached to the backbone molecules such as hydroxylation, methoxylation, isoprenylation, and glycosylation [29]. Flavones are commonly consumed from vegetables, fruits, and herbs, such as parsley, celery, chamomile, and marjoram, and from olives and extra virgin olive oil. Major flavones include apigenin, luteolin, tangeretin, eupatilin, jaceosidin, baicalein, and wogonin [30]. The daily flavone intake of US adults is 1.6 ± 0.2 mg/day based on the National Health and Nutrition Examination Survey (NHANES) 1999–2002 [31].

#### 2.1.2. Effects of Flavones on Non-Shivering Thermogenesis

Luteolin (2-(3,4-dihydroxyphenyl)-5,7-dihydroxy-4-chromenone) is found in carrots, peppers, celery, olive oil, peppermint, thyme, rosemary, and oregano [32]. A supplementation of 0.01% luteolin increased the expression of the *Ucp1*, *Ppgc1α*, *Pparα*, *Cidea,* and *Sirt1* genes, which are involved in the regulation of non-shivering thermogenesis in the BAT of high-fat diet (HFD)-fed mice (45% calories from fat, 12 weeks), as was further evidenced by denser multilocular and smaller lipid droplets [33]. However, the expression of *Prdm16* and the elongation of very-long chain fatty acids-like 3 (*Elovl3*) genes was not changed [33]. Together with markers for beige adipocytes, *Tmem26*, *Cd137*, and *Cited1* genes were also induced in subcutaneous WAT (SWAT). Consistent with the increased expression of genes related to non-shivering thermogenesis in SWAT, luteolin supplementation reduced fat mass and body weight through an increase in energy metabolism, as confirmed by improvements in oxygen consumption, carbon dioxide production, and respiratory exchange rate, compared to HFD-fed mice [33]. Luteolin caused AMPK phosphorylation, which increased the activity of SIRT1 to enhance the protein expression and activity of PGC1α and increased the phosphorylation of ACC that turns on energy metabolism toward AMPK-activated energy expenditure. Because SIRT1 and PGC1α are essential regulators for mitochondrial biogenesis and function, luteolin thus increased non-shivering thermogenesis via the AMPK/SIRT1/PGC1α pathway [33,34,35].

Chrysin (5,7-dihydroxyflavone), which is present in high amounts in honey, propolis, and honeycomb, showed antidiabetic, anti-inflammatory, and anticancer effects [36,37,38]. Chrysin (50 µM) exhibited promising effects on non-shivering thermogenesis by inducing 3T3-L1 cells to exhibit the phenotype of brown adipocytes [39]. Although 3T3-L1 cells are fully committed to undergoing differentiation toward the white adipocyte phenotype, 3T3-L1 preadipocytes can also be induced to differentiate into beige-like adipocytes through prolonged treatment with beige induction cocktails (dexamethasone, 3-isobutyl-1-methylxanthine, insulin, triiodothyronine, and rosiglitazone), and partly through the manipulation of C/EBPβ [40,41]. As part of the beige cocktail treatment, chrysin induced the expression of beige specific genes such as *Tbx1*, *Tmem26*, and *Cited1*, and genes that are involved in the regulation of brown adipogenesis such as *Ucp1* and *Prdm16*, *Pgc1α, C/ebpβ,* and *Fgf* 21, and the *Cidea* gene, which is related to the development of lipid droplets [39]. Together with the browning of 3T3-L1 adipocytes, chrysin also increased the expression of genes and proteins related to lipolysis, namely, HSL, perilipin (PLIN), acyl-coenzyme A oxidase (ACO), and carnitine palmitoyltransferase 1 alpha (CPT1α). The cells that were treated with the AMPK inhibitor, compound C, or the AMPK activator 5-aminoimidazole-4-carboxamide ribonucleotide showed decreased and increased PRDM16, UCP1, and PGC1α protein levels, respectively, which indicated AMPK-mediated regulation of chrysin [39].

Olive (*Olea europaea*) leaf extract (OLE) contains flavones, such as luteolin and apigenin [42]. HFD-fed mice (40% calories from fat) supplemented with 0.15% OLE for 8 weeks showed the induced expression of genes involved in mitochondrial biogenesis, such as mitochondrial transcription factor A (*Tfam*), nuclear respiratory factor (*Nrf*)1, and cytochrome C oxidase, and in non-shivering thermogenesis, such as *Sirt1*, *Pgc1α*, and *Ucp1* in epididymal WAT (EWAT), indicating the browning effect [43]. OLE also decreased food intake and the expression of *Pparγ*, *C/ebpα*, *Cd36*, and fatty acid synthase (*Fas*) genes that are involved in the regulation of adipogenesis and fatty acid synthesis. These changes caused a reduction in body weight, visceral fat pad (epididymal, perirenal, retroperitoneal, and mesenteric) weight, and plasma concentrations of triglyceride, total cholesterol, very-low density lipoproteins (VLDL), LDL, and free fatty acids [43]. Interestingly, the decreased white adipogenesis was associated with two mechanisms that are involved in the development of white adipocytes, the wingless type (WNT) and the galanin-mediated signaling pathway. OLE activated the WNT signaling pathway via increasing the WNT MMTV integration site family, member 10b (*Wnt10b*) and low-density lipoprotein receptor-related protein-5 genes and decreasing secreted frizzled-related protein 5. However, Lo et al. indicated that WNT inhibition using the WNT inhibitor C59 caused white adipocytes browning [44]. Genes that were involved in the galanin-mediated signaling pathway, such as galanin, galanin receptor 1 and 2, PKCδ, and cyclin D, were downregulated together with decreased phosphorylation of extracellular signal-regulated kinase, which further caused the reduced expression of adipogenic and lipogenic genes, such as *Pparγ*, *C/ebpα*, *Cd36*, and *Fas* [43].

Intestinal bacteria are critical for regulating the bioavailability of flavonoids, which would further affect the biological effect of flavonoids in the body [45]. Germ-free or antibiotic treatments might promote the generation of a population of bacteria that can degrade flavonoids, which would affect flavonoid-induced non-shivering thermogenesis [46,47,48]. Supplementation with a mixture of apigenin (4′,5,7-trihydroxyflavone) and naringenin (4′,5,7-trihydroxyflavanone) (80 mg/kg), which is a flavanone that is predominantly found in grapefruit, increased *Ucp1* gene expression in the BAT of mice administered antibiotics during weight-cycling intervention [46]. As demonstrated through an indirect calorimetry challenge, these mice also showed a higher energy expenditure than mice that were not treated with antibiotics during this intervention and mice fed an HFD during the entire experimental period [46].

Sudachitin (5,7,4′-trihydroxy-6,8,3′-trimethoxyflavone) is a polymethoxylated flavone that is found in the Japanese citrus fruit sudachi [49]. Mice fed a HFD (40% of calories from fat) that received a daily oral administration of 5 mg/kg sudachitin for 12 weeks displayed lower body weight, improved glucose tolerance, and better insulin sensitivity compared with control mice [50]. The effects of sudachitin were also observed in ob/ob mice, but these mice did not exhibit a change in body weight. The administration of sudachitin increased energy expenditure in both mice, but this increase was not correlated with non-shivering thermogenesis, a function of BAT, as evidenced by a lack of change in UCP1 expression in BAT. Although *Ucp1* gene expression was increased in SWAT, the sudachitin-induced increase in energy expenditure was supported by increased mitochondrial function in muscle, which showed upregulated expression of the *Sirt1*, *Pgc1α*, *Nrf1*, *Nrf2*, *Tfam*, *Pparα*, *Ucp2,* and *Ucp3* genes [50], Table 1**.**

### 2.2. Flavonols

#### 2.2.1. Major Food Sources and Daily Intake

Flavonols are a group of flavonoids with a 3-hydroxy-2-phenylchromen-4-one backbone. Naturally occurring flavonols include quercetin (3,5,7,3′,4′-pentahydroxyflavone), myricetin (3,5,7,3′,4′,5′-hexahydroxyflavone), kaempferol (3,5,7,4′-tetrahydroxyflavone), isorhamnetin (3,5,7,4′-tetrahydroxy-3′-methoxyflavone), and rutin (quercetin-3-O-rutinoside). The major food sources of flavonols are kale, onions, apples, teas, buckwheat, and broccoli [51]. The daily flavonol intake of US adults was 12.9 ± 0.4 mg/day based on the NHANES 1999–2002 [31].

#### 2.2.2. Effect of Flavonols on Non-Shivering Thermogenesis

Onion peel, a byproduct of onion, contains high concentrations of quercetin, which is the most abundantly consumed flavonol. Onion peel extract (OPE) and quercetin exerted anti-obesity effects by inhibiting adipogenesis and lipogenesis [52,53]. In an *in vitro* study using 3T3-L1 cells, OPE (75 µg/mL and 100 µg/mL) reduced the expression of the adipocyte protein 2 (*Ap2*) and *Acc* genes and, interestingly, increased the expression of the *Cpt1α* gene, which encodes a rate-limiting enzyme in the beta-oxidation of fatty acids [52]. Consistent with this finding, Sprague-Dawley rats fed an HFD (40% calories from fat) supplemented with 0.72% OPE showed decreased *Pparγ*, *C/ebpα*, *Fas*, and *Acc* gene expression and increased expression of the *Cpt1α* and *Ucp1* genes in EWAT [52]. However, 0.5% OPE significantly induced *Ucp1*, *Prdm16*, *Pparγ*, and *Cidea* expression in retroperitoneal WAT (RWAT) and did not upregulate these genes in the EWAT and SWAT of mice fed an HFD (60% calories from fat) [54]. This browning effect was in part mediated by the AMPK/SIRT1/PGC1α signaling pathway [54]. Notably, the AMPK/SIRT1/PGC1α signaling pathway was also critical for the quercetin-regulated anti-inflammatory effect [53]. Consistent with the activation of fat utilization in WAT [54], mice fed a HFD (45% calories from fat) supplemented with 0.1% quercetin showed improvements in the physiological function of WAT [53]. These changes were mediated by the amelioration of macrophage infiltration and inflammatory cytokine markers, and increased *Ucp1* gene expression in BAT, which suggested increased energy expenditure [53]. Although energy expenditure was not clearly increased by quercetin, this flavonol increased the physical activity of mice by increasing the mitochondrial number in skeletal muscle and increasing the expression of genes encoding polypeptides for subunits of the mitochondrial complexes that possibly have a higher oxidative capacity [55,56]. Interestingly, isoquercetin (quercetin-3-glucoside), which is also found in onion peel, was not found to exert the adipocyte browning effect in an *in vitro* study [54].

Rutin (quercetin-3-rutinoside) (1 mg/mL), the other glycoside of quercetin, increased energy expenditure by inducing BAT activity and BAT- and beige adipocyte-specific genes in the BAT and WAT of both ob/ob and C57Bl/6J mice with HFD (60% calories from fat)-induced obesity [57]. To promote non-shivering thermogenesis in BAT and WAT, the expression of genes involved in the regulation of lipolysis that provides free fatty acids were also increased. Mitochondrial biogenesis, as evidenced by increased *Tfam*, *Nrf1*, and *Nrf2* gene expression, was further enhanced by rutin. PGC1α is a well-known transcriptional factor that is required for the regulation of non-shivering thermogenesis [58]. PGC1α-expressing mesenchymal stem cells showed significantly increased mitochondrial biogenesis and respiration [59]. Consistent with this finding, rutin-induced non-shivering thermogenesis was attributed to SIRT1 stabilization-activated PGC1α deacetylation [57]. Based on the beneficial effect of rutin to activate thermogenesis through BAT, the therapeutic potential of rutin was studied in dehydroepiandrosterone (DHEA)-induced polycystic ovary syndrome (PCOS) rats [60]. DHEA-induced PCOS rats showed decreased expression of BAT-specific genes and genes that are involved in the regulation of lipid metabolism, such as *Ucp1* and *Pparα*, medium-chain acyl-coenzyme A (*Mcad*), *Pgc1α*, *Pgc1β*, and *Cpt1α* genes and reduced expression of the UCP1 protein and other proteins that are involved in the regulation of oxidative phosphorylation such as ATP synthase F1 subunit alpha (ATP5α), ubiquinol-cytochrome c reductase core protein 2 (UQCRC2), and succinate dehydrogenase complex iron sulfur subunit B (SDHB), which decreased heat generation in BAT. This molecular and physiological reduction was reversed by rutin treatment to the normal level of a control group of rats that were not treated with DHEA. Consistent with increased BAT function, DHEA-induced PCOS rats supplemented with rutin showed increased insulin sensitivity, a critical factor to improve the POCS condition compared to the DHEA-induced PCOS rats [60]. Therefore, rutin may rescue PCOS infertility by increasing energy expenditure through BAT and further enhancing insulin sensitivity [60].

Other flavonols, namely, myricetin and kaempferol, also showed the potential to increase energy expenditure [61,62]. Myricetin supplementation (400 mg/kg) increased BAT activity and the expression of *Ucp1*, *Sirt1*, *Atp5α*, and *Pgc1α* genes in BAT and caused browning effect on inguinal WAT (IWAT) by increasing the expression of the *Tbx1*, *Cd137*, and *Tmem26* genes in db/db mice [61]. Together with increased mitochondrial DNA copy number, it indicated that myricetin-induced non-shivering thermogenesis may result from increased mitochondrial biogenesis through SIRT1-induced deacetylation and PGC1α activity. Increased energy expenditure via myricetin reduced the body weight and fat mass of db/db mice and improved the plasma lipid profile and other obesity-related metabolic alterations, such as glucose tolerance, insulin resistance, and hepatic steatosis [61]. Kaempferol increased the expression of the *Pgc1α*, *Cpt1α*, *Tfam*, citrate synthase, and *Ucp3* genes in human skeletal muscle myocytes in vitro and increased DIO2 activity, which regulates the thyroid hormone, a major metabolic hormone, and thereby potentially increased energy expenditure [62].

### 2.3. Flavanones

#### 2.3.1. Major Food Sources and Daily Intake

The flavanone structure is 2,3-dihydro-2-phenylchromen-4-one [63]. Naturally occurring flavanones include naringenin (5,7,4′-trihydroxyflavanone), hesperetin (5,7,3′-trihydroxy-4′-methoxyflavanone), eriodictyol (5,7,3′,4′-tetrahydroxyflavanone), narirutin (naringenin-7-O-rutinoside), hesperidin (hesperetin 7-O-rutinoside), and eriocitrin (eriodictyol 7-O-rutinoside) [64]. As a major food source, citrus fruits, such as satsuma mandarin orange (*Citrus unshiu* Marc.) and valencia orange (*Citrus sinensis* Valencia), contain narirutin and hesperidin [65]. The daily flavanone intake of US adults is 14.4 ± 0.6 mg/day, as demonstrated by the NHANES 1999–2002 [31].

#### 2.3.2. Effect of Flavanones on Non-Shivering Thermogenesis

Extract from hesperidin-rich *Gelidium elegans* (20 and 40 µg/mL), a red seaweed, reduced lipid accumulation during 3T3-L1 cell differentiation due to decreased *Pparγ* and *Ap2* expression [66]. *Gelidium elegans* (5%) caused anti-adipogenesis due to increased lipolysis in the EWAT of streptozotocin-nicotinamide-induced diabetic rats, which suggested the potential of *Gelidium elegans* to increase energy expenditure [67]. Consistent with accelerated lipolysis [67], the extract increased PRDM16 and UCP1 protein expression in the BAT of HFD-induced obese mice by activating the AMPK pathway [68]. Although 3T3-L1 cells treated for 8 days with 20 µM hesperidin, a major dietary compound of *Gelidium elegans*, showed decreased triglyceride synthesis and increased expression of the adipose triglyceride lipase gene, *Gelidium elegans* extract showed stronger effects on the induction of *Ucp1* and *Prdm16* expression [69]. The oral administration of 4G-α-glucopyranosyl hesperidin, a water-soluble form of hesperidin, also increased body temperature by activating BAT [70].

### 2.4. Flavanols

#### 2.4.1. Major Food Sources and Daily Intake

The backbone structure of flavanols is 2-phenyl-3,4-dihydro-2H-chromen-3-ol, which is referred to as flavan-3-ol [71]. Interestingly, the major intake of flavonoids in the US originates from the intake of flavanols [72]. A study using data from the NHANES 1999–2002 indicated that the average daily intake of flavan-3-ols is 156.5 ± 11.3 mg/day, which accounts for 82% of the total flavonoid intake [31]. Although flavanols are commonly found in tea, wine, apples, and chocolate, black tea is a major contributor to the consumption of flavan-3-ols in the American diet [72,73]. The monomers catechin, epicatechin, epigallocatechin (EGC), epicatechin gallate (EG), and epigallocatechin gallate (EGCG), and oligomeric proanthocyanidin are classified as flavanols [74].

#### 2.4.2. Effects of Flavanols on Non-Shivering Thermogenesis

Green tea (*Camellia sinensis*) is recognized as a weight loss booster by increasing energy expenditure and thermogenesis due to the presence of caffeine and catechins [75]. The administration of green tea extract (20 g/kg diet) reversed HFD-reduced energy expenditure of Sprague-Dawley rats with HFD-induced obesity to the same level of energy expenditure as observed in rats fed a normal diet [76]. This effect was inhibited by the β-AR antagonist propranolol, which indicates a critical role of β-AR in green tea-mediated energy expenditure [76]. Nomura et al. reported that 0.5% green tea catechins increased *Ucp1* gene expression in the BAT of rats fed a chow diet (5% fat) but not in rats fed an HFD (35% fat) compared with their control groups [77]. Consistent with the studies using rats, mice fed both chow and an HFD supplemented with green tea catechins (100 mg/kg) showed upregulated expression of the *Ucp1*, *Cpt1α,* and *Aco* genes in BAT [76,77,78]. The expression of the *Ucp1* gene was also induced in SWAT and visceral WAT in a manner consistent with increased and decreased *Pparγ* expression, respectively [78]. One of the necessary hormones for activating non-shivering thermogenesis in BAT is norepinephrine, which stimulates G-protein coupled β_3_-AR [79]. Catechol-P-methyltransferase (COMT) is an enzyme that degrades norepinephrine by catalyzing the addition of a methyl group to norepinephrine to generate normetanephrine [80]. EGCG and EGC acted as substrates for inhibiting COMT activity [81]. This indicated a relationship between EGCG and sympathetic activity. Green tea with high concentrations of EGCG and caffeine or EGCG independently increased the respiration rate of interscapular BAT, whereas caffeine did not affect the respiration rate [75]. This green tea-induced respiration rate was further stimulated by ephedrine, an enhancer of norepinephrine. These effects were not shown in interscapular BAT obtained from chemically sympathectomized rats. Although caffeine independently could not change the respiration rate, EGCG’s effect on the respiration rate was synergistically induced in a dose-dependent manner (50, 100, and 200 µM), together with 100 M caffeine under 0.25 µM ephedrine [75]. This boosting effect on the respiration rate by the combination of EGCG and caffeine may result from the effect of caffeine on the protection of norepinephrine-β-AR-induced cAMP by inhibiting phosphodiesterase, an enzyme that degrades cAMP [75]. Compared to green tea, oolong tea, black tea, and pu-erh tea also showed the potential to increase energy expenditure, possibly by increasing AMPK phosphorylation in the mesenteric WAT, SWAT, and BAT of mice, which would result in the reduction of adiposity [82]. However, UCP1 protein expression was only significantly increased in the WAT of mice that ingested black tea, indicating that the degrees of oxidation and fermentation and the flavanol composition of tea might be critical factors for tea-induced non-shivering thermogenesis [82].

The effect of green tea extract on energy expenditure through non-shivering thermogenesis has been further evidenced in clinical studies [83,84,85]. Healthy young men who were administered green tea extract consisting of 50 mg of caffeine and 90 mg of EGCG showed increased energy expenditure, respiratory quotient, and urinary norepinephrine excretion without any urinary excretion of nitrogen [83]. None of these changes were observed in a group that ingested caffeine alone, which suggested that green tea-induced non-shivering thermogenesis might be mediated by catechins and not by caffeine [83]. In a double-blinded design, healthy young women who drank a catechin-rich beverage (540 mg/day of catechin intake) for 12 weeks exhibited an 18.8% increased BAT density and a 17.4% lower amount of extramyocellular lipids than a placebo group, as detected by near-infrared spectroscopy [84]. When exposed to a cold water (15 °C) environment for 3 h, healthy male subjects who ingested 1600 mg of EGCG and 600 mg of caffeine from green tea showed a 10% higher energy expenditure and a 20% lower shivering intensity than subjects who ingested a placebo [85]. 

Another flavanol source that exerts a positive effect on non-shivering thermogenesis is chocolate, which is enriched in flavan-3-ols such as (+)-catechin, (−)-epicatechin, and B-type procyanidins [86,87,88]. Mice administered a dose of 10 mg/kg of a flavan-3-ol fraction composed of 4.56% (+)-catechin, 6.43% (−)-epicatechin, 3.93% procyanidin B2, 2.36% procyanidin C1, and 1.45% cinnamtannin A2 showed increased expression of *Ucp1* and *Pgc1α* genes in BAT and *Ucp3* and *Pgc1α* genes in the gastrocnemius, a higher resting energy expenditure, and increased plasma adrenaline concentrations [86]. However, these changes were not observed in mice supplemented with (−)-epicatechin [86]. Pretreatment with butaxamine, a β_2_-AR blocker, and/or SR59230, a β_3_-AR blocker, prevented all flavan-3-ol fraction-induced changes, except for the increase in AMPK phosphorylation in the gastrocnemius muscle in the presence of a β_3_-AR blocker [87]. This indicated that the induction of non-shivering thermogenesis by flavan-3-ol was mediated by the activation of AMPK1α through its phosphorylation.

Rabadan-Chávez et al. showed that cocoa powder (10 mg/mL) and cocoa extract (10 mg/mL) upregulated the expression of *Pparα*, *Pgc1α, Sirt1,* and *Ucp1* genes, which caused browning effect on the RWAT of rats with HFD-induced obesity, but this upregulation was less effective than that induced individually by the flavanols epicatechin, catechin, and procyanidin B2 [88]. *Pparγ* and *Cd36* genes were upregulated whereas the expression of *Acc* gene was downregulated by these extracts. [88]. Consistent with these changes, the HFD-induced concentrations of plasma lipids were improved by the cocoa-derived products. Although a mixture of flavan-3-ols, such as cocoa powder or cocoa extract, had a higher impact on the activation of non-shivering thermogenesis, (-)-epicatechin significantly induced adipocyte browning and improved mitochondrial function by increasing the expression of the SIRT1, PGC1α, SIRT3, UCP1, and DIO2 proteins in the EWAT of rats fed an HFD [89].

### 2.5. Anthocyanins

#### 2.5.1. Major Food Sources and Daily Intake

As a type of flavonoid, anthocyanins are water-soluble pigments that provide blue, purple, and red colors in fruits and vegetables, such as berries, grapes eggplant, and red cabbage [90]. The structure of anthocyanins contains a backbone of 2-phenylchromenylium [91]. These flavonoids are generated by the addition of various sugar molecules to the structure of the major anthocyanidins, namely, peonidin pelargonidin, malvidin, cyanidin, petunidin, and delphinidin [92]. Given their various health benefits, such as antioxidant, anti-inflammation, anti-diabetes, anti-cancer, and anti-cardiovascular disease properties, the consumption of foods containing anthocyanins is highly recommended [90]. The daily intake of anthocyanins in the US, as demonstrated by the NHANES 2001–2002, is 3.1 ± 0.5 mg/day [31].

#### 2.5.2. Effect of Anthocyanins on Non-Shivering Thermogenesis

Cyanidin-3-glucoside, which is also called chrysanthemin, is the most abundantly ingested anthocyanin [93]. When C_3_H_10_T_1/2_ mesenchymal stem cells were treated with mulberry extract (ME) and mulberry wine extract (MWE) (10 µg/mL), which contained approximately 69.93 ± 1.31 and 8.50 ± 0.12 mg/g dry weight of cyanidin-3-glucoside, respectively, the expressions of the *Ucp1*, *Pgc1α*, *Prdm16*, and *Cpt1α*, genes were increased without changes in genes that are involved in the regulation of adipogenesis, e.g., *Ap2*, *C/ebpα*, *C/ebpβ*, and *Pparγ2* during brown adipogenesis [94]. Consistent with increased expression of the *Tfam* and *Nrf*-*1* genes, both ME and MWE increased the mitochondrial number and the expression of mitochondrial-specific proteins, e.g., UQCRC2 and 246 NADH dehydrogenase (ubiquinone) 1 beta subcomplex, 8 (NDUFB8) in C_3_H_10_T_1/2_ cells. The cells treated with ME, but, not MWE showed increased UCP1 protein expression. The β_3_-AR agonist (CL316,2432) stimulated the phosphorylation of p38 mitogen-activated protein kinase (MAPK) by protein kinase A in both white and brown adipocytes, which led to an increase in non-shivering thermogenesis and mitochondria biogenesis [95]. Thus, ME-induced UCP1 expression may result from the upregulated phosphorylation of p38 mitogen-activated protein kinases, which might be critical modulators of brown adipocyte development [94]. Takikawa et al. reported that KK-A^y^ mice supplemented with bilberry extract (27 g/kg diet, the extract contains 375 g of anthocyanins/kg) significantly activated AMPK in WAT and skeletal muscle [96].

Consistent with the results of studies performed with whole extracts of anthocyanin-rich-foods, the treatment of 3T3-L1 cells with 50 µM or 100 µM cyanidin-3-glucoside also converted 3T3-L1 preadipocytes to beige adipocytes during differentiation [97]. A cyanidin-3-glucoside-induced browning effect was possibly due to both reduced AMP/ATP level-induced AMPK activation and enhanced brown adipogenesis by cAMP-mediated C/EBPβ, a transcriptional factor that is involved in the adipogenesis induced PGC1α-UCP1 pathway [97]. The db/db mice that were administered drinking water containing cyanidin-3-glucoside (1 mg/mL) for 16 weeks showed beige cell formation in SWAT and increased BAT activity [98]. These mice showed weight loss and decreased SWAT and EWAT weights, compared to that in a control group that was administered drinking water without the addition of cyanidin-3-glucoside [98].

### 2.6. Isoflavones

#### 2.6.1. Major Food Sources and Daily Intake

Isoflavones are known as phytoestrogens, because they mimic human estrogen activity [99], and their structures have a 3-phenylchromen-4-one backbone [100]. As primary food sources of isoflavones, soy, soy products, red clover, and kudzu have high amount of isoflavones, such as daidzein, genistein, glycitein, biochanin A, and formononetin [101]. The isoflavone intake in the US is estimated as 1.2 ± 0.2 mg/day based on the NHANES 1999–2002 [31].

#### 2.6.2. Effect of Isoflavones on Non-Shivering Thermogenesis

Estrogen is a hormone that plays a role in developing female characteristics, but it is also involved in the regulation of energy balance. Postmenopausal women and ovariectomized mice showed decreased levels of 17β-estradiol (E2), a major estrogen that is associated with weight gain and increased fat content in the body [102]. Estradiol may increase energy metabolism by increasing BAT-mediated non-shivering thermogenesis through the inhibition of AMPK in the sympathetic nervous system [103]. Although various studies have focused on the phytoestrogenic function of isoflavones, their potential to increase non-shivering thermogenesis was recently discovered [104,105,106,107,108]. The isoflavone-rich fraction of the Kudzu (*Puerariae thomsonii*) flower exerted anti-obesity effects by increasing lipolysis in WAT and *Ucp1* expression in the BAT of mice with HFD-induced obesity [104]. *Ucp1* mRNA expression was also significantly increased in the BAT of male Long-Evans rats administered a soy-based isoflavone-rich diet (600 µg/g of diet) compared to that in rats fed a low amount of isoflavones (10–15 µg/g of diet) [105].

Consistent with data from studies using isoflavone-rich food extracts, isoflavones also showed the potential to induce non-shivering thermogenesis in animal and white adipocytes [104]. Daidzein supplementation (50 mg/kg) for 14 days reduced body weight by ameliorating hepatic lipid accumulation, and this reduction was achieved by decreasing stearoyl coenzyme A desaturase 1, which is involved in the regulation of lipogenesis [106]. UCP1 protein expression in BAT was also increased [106], which suggested daidzein-induced non-shivering thermogenesis. In a study using 3T3-L1 cells, treatment with genistein (50 or 100 µM) induced a switch in the phenotypes of white adipocytes toward BAT-like adipocytes during adipogenesis [107]. These adipocytes showed the decreased expression of lipogenic and adipogenic genes and increased the expression of the *Ucp1* gene and multilocular lipid droplets [107]. Accompanying this change, oxygen consumption was enhanced at both the basal and the maximal respiratory capacity. Using SIRT1 or an estrogen receptor inhibitor, Aziz et al. found that the genistein-induced browning effect was due to SIRT1 and not to an estrogen receptor-regulated pathway [107]. Formononetin (10 nM) significantly inhibited mRNA and protein expression of transcription factors that regulate adipogenesis, including PPARγ, C/EBPα, and sterol regulatory element-binding protein 1 [108]. An *in vivo* study using mice with HFD-induced obesity also supported the anti-adipogenesis and browning effects of formononetin [108]. PPARγ and C/EBPα protein expression was significantly reduced, whereas the expression of the UCP1, ELOVL3, and DIO2 proteins was induced in the WAT of mice supplemented with formononetin by activating the AMPK/β-catenin signaling pathway [108], Table 2 and Table 3.

## 3. Conclusions

**Figure 2:**
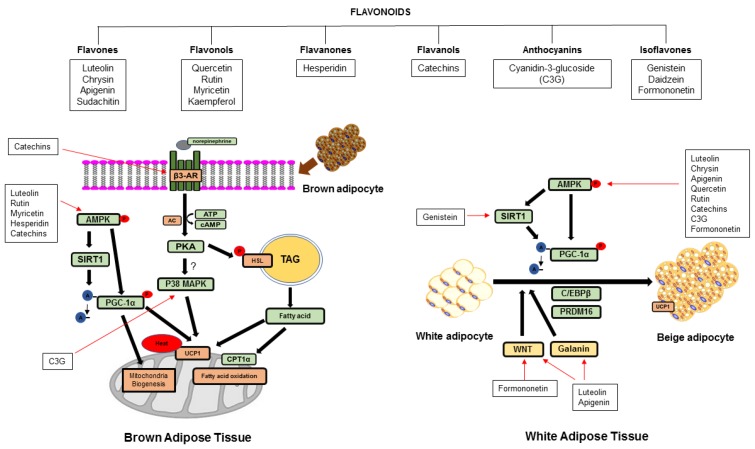
Suggested mechanistic pathway of different flavonoids in the activation of non-shivering thermogenesis in BAT and WAT. AC; adenylyl cyclase, AMPK; AMP-activated protein kinase, ATP; adenosine triphosphate, β-AR; beta-adrenergic receptor, cAMP; cyclic AMP, C/EBPβ; CCAAT/enhancer-binding protein beta CPT1α; carnitine palmitoyltransferase 1 alpha, HSL; hormone sensitive lipase, PGC1α; peroxisome proliferator-activated receptor gamma coactivator 1 alpha, PKA; protein kinase A, PRDM16; positive regulatory domain containing 16, p38 MAPK; p38 mitogen-activated protein kinase, SIRT1; silent mating type information regulation 2 homolog 1, TAG; triacylglycerol, UCP1; uncoupling protein 1, WNT; wingless type.

Increasing non-shivering thermogenesis by BAT activation and WAT browning is a potential approach for improving metabolic diseases. As shown in Figure 2, most dietary compounds except sudachitin increased the expression of the UCP1 gene or protein, which suggested increasing non-shivering thermogenesis. Flavonoids may regulate UCP1-mediated non-shivering thermogenesis by (i) the AMPK-activated SIRT1-PGC1α pathway, which possibly induces mitochondrial biogenesis and lipolysis and fatty acid oxidation and (ii) regulating PRDM16 in the complexed regulation with PPARγ, C/EBPα and β, PGC1α, and SIRT1 during differentiation. Fiorani et al. showed that quercetin was taken up and then stored in the mitochondria and was re-distributed into the cytosol to prevent cell damage by reactive oxygen species [109]. Therefore, flavonoids may be taken up into the mitochondria, which directly regulates thermogenic proteins. PGC1α is localized in the cytosol and nucleus depending on its activation, whereas SIRT1 is localized in the cytosol [110,111]. Various flavonoids showed increased PGC1α and deacetylation on PGC1α and SIRT1. Therefore, the stored flavonoid in mitochondria may be re-distributed into other subcellular compartments, e.g., the cytosol, which regulates the deacetylation of PGC1α by SIRT1. Flavonoids may cause SIRT1-activated deacetylated PPARγ to efficiently bind to PRDM16, which promotes brown adipogenesis and the browning effect, which is similar to the action of thiazolidinedione [112]. As uncouplers, flavonoids may regulate non-shivering thermogenesis. 2.4-dinitrophenol (DNP), an uncoupler, increases non-shivering thermogenesis by a proton leakage-diminished proton gradient that causes uncoupling oxidative phosphorylation and prevents the uptake of inorganic phosphate into mitochondria, causing reduced ATP synthesis [113]. However, some flavonoids may not function as uncouplers.

The current review paper has some limitations. Although the authors searched for literature that studied the effects of flavonoids, most of the literature shown in the current paper exhibited a positive effect of flavonoids on non-shivering thermogenesis. Some dietary flavonoids were effective at the clinical levels, but most of the data show flavonoids’ effects on BAT and browning WAT at pre-clinical levels using mammalian cells and animals, e.g., mice and rats. These preclinical studies may not have the same clinical effects. It may be difficult to measure BAT activity by ingested flavonoids in humans. Recently, infrared thermography showed the same results as positron emission tomography-computed tomography scan, which avoids radiation [114]. Therefore, clinical studies should be performed to confirm flavonoids’ effect on non-shivering thermogenesis using infrared thermography before the flavonoids are suggested for improving metabolic diseases. Although flavonoids have shown a positive effect on energy metabolism by regulating non-shivering thermogenesis, major concerns of flavonoids include the low bioavailability when flavonoids are ingested and their structure modification through the digestive system. On the surface of epithelial cells of the small intestine, flavonoids undergo de-glycosylation by lactase-phlorizin hydrolase [115,116]. Subsequently, de-glycosylated flavonoids are absorbed through sodium-glucose co-transporter 1 and glucose transporter (GLUT) 2 on the epithelial cells of small intestine [117,118]. The flavonoids absorbed from intestinal lumen undergo glucuronidation by UDP-glucuronosyl transferase as the detoxifying mechanism, called phase II detoxification [119,120]. The absorbed metabolites can be transferred to the liver through portal circulation. In the liver, flavonoid metabolites are further metabolized for phase II detoxification by methylation, sulfation, and glucuronidation [121]. These metabolites are circulated and distributed into several tissues. Although there is no clear evidence that ingested intact flavonoids are transferred and taken up into adipose tissues, GLUT 4, which is expressed in adipose and muscle cells, has been identified as a possible transporter for flavonoids, such as genistein, myricetin, and quercetin [122]. Some flavonoids are metabolized by intestinal bacteria in the large intestine and then absorbed into the body as well [123]. This suggests that the effects of flavonoids on non-shivering thermogenesis may be regulated by indirect signaling cascades such as the microbiome.

Taken together, future studies to understand the effects of flavonoids on non-shivering thermogenesis should be performed together with determining bioavailability of flavonoids and considering indirect regulation through the microbiome at the clinical level.

## Figures and Tables

**Table 1:** Flavonoid intake of U.S. adults aged 19+ y based on the NHANES 1999–2002 database.

Groups	Dietary Flavonoid Intakes (mean ± SD) ^a^
Flavone	1.6 ± 0.2 mg/day
Flavonol	12.9 ± 0.4 mg/day
Flavanol	156.5 ± 11.3 mg/day
Flavanone	14.4 ± 0.6 mg/day
Anthocyanin	3.1 ± 0.5 mg/day
Isoflavone	1.2 ± 0.2 mg/day

^a^ Dietary flavonoid intakes of US adults (19+ years) were estimated based on the 24-hour-dietary recall of National Health and Nutrition Examination Survey 1999–2002 (*n* = 8809).

**Table 2:** *In vitro* evidence for increase of non-shivering thermogenesis by flavonoids.

Flavonoids		Subjects	Treatments	Outcomes	Authors
Flavone	Luteolin	Primary adipocytes from BAT and SWAT	100 nM	↑ *Ucp1*, *Pgc1α* and *Sirt1* in BAT and SWAT ↑ AMPK phosphorylation in BAT and SWAT	Zhang et al. [33]
	Chrysin	3T3-L1	50 µM	↑ UCP1, *Ucp1*, PGC1α, *Pgc1α* PRDM16, *Prdm16*, FGF21, *Fgf21*, TBX1, *Tbx1*, TMEM26, *Tmem26*, CIDEA, *Cidea* and CITED 1, *Cited1* ↑ AMPK phosphorylation	Choi et al. [39]
	Sudachitin	Primary myoblasts	30 mM	↑ *Sirt1*, *Pgc1α*, and *Ucp1*	Tsutsumi et al. [50]
Flavonol	Onion peel (quercetin)	3T3-L1	25-100 µg/mL	↑ *Cpt1α*, *Fabp4*	Moon et al. [52]
	Quercetin	3T3-L1	25–100 µM	↑ *Ucp1*, *Cpt1α*, *Tbx1*, *Pgc1α*, *Ppar*γ, and *Prdm16*	Lee et al. [54]
	Rutin	C_3_H_10_T_1/2_ cells	0.1–100 µM	↑ UCP1, *Ucp1, Prdm16*, *Pgc1α*, *Tfam*, *Nrf1,* and *Nrf2* ↑ PGC1α deacetylation by stabilizing SIRT1	Yuan et al. [57]
	Myricetin	C_3_H_10_T_1/2_ cells	0.001–10 µM	↑ *Ucp1*, UCP1, PGC1α, SIRT1, and adiponectin	Hu et al. [61]
Flavanone	Gelidium elegans (Hesperidin)	3T3-L1	12.5 and 50 µg/mL	↑ UCP1 and PRDM16	Choi et al. [69]
Anthocyanin	Mulberry extract (ME), mulberry wine extract (MWE) and cyanidin-3-glucoside (C3G)	C_3_H_10_T_1/2_ cells	ME and MWE (10 µg/mL) of C3G (1–100 µM)	↑ UCP1, *Ucp1*, *Pgc1α*, *Cpt1α*, and *Prdm16* by ME and MWE ↑ Phosphorylation of p38 MAPK by ME ↑ UCP1, *Ucp1*, *Pgc1α*, *Pgc1β*, *Prdm16*, *Nrf1*, mitochondrial copy number, and cellular oxygen respiration by C3G	You et al. [94]
	C3G	3T3-L1	50 or 100 µM	↑ Cellular cAMP concentration ↑ AMPK phosphorylation ↑ FABP4, UCP1, PGC1α expression ↑ Mitochondrial biogenesis ↑ *C/ebpβ*, *Tbx1* and *Cited 1*	Matsukawa et al*.* [97]
Isoflavone	Genistein	3T3-L1	100 µM	↑ *Ucp1*, *Pgc1α*, and *Sirt1* ↑ Oxygen consumption	Aziz et al. [107]
	Formononetin	3T3-L1	10 nM	↑ AMPK phosphorylation and *β*-catenin expression	Gautam et al. [108]

AMPK; 5′ AMP-activated protein kinase, BAT; brown adipose tissue, C/EBPβ; CCAAT/enhancer-binding protein beta, cAMP; cyclic AMP, CIDEA; cell death-inducing DFFA-like effector a, CITED 1; Cbp/p300-interacting transactivator, CPT1α; carnitine palmitoryl transferese 1 alpha, ELOVL3; elongation of very-long chain fatty acids-like 3, FABP4; fatty acid binding protein 4, FGF21; fibroblast growth factor 21, NRF1 or 2; nuclear respiratory factor 1 or 2, PGC1α; PPARγ coactivator 1 alpha, PRDM16; positive regulatory domain containing 16, PPARα; peroxisome proliferator-activated receptor alpha, PPARγ; peroxisome proliferator-activated receptor gamma, p38 MAPK; p38 mitogen-activated protein kinase, SIRT1; silent mating type information regulation 2 homolog 1, SWAT; subcutaneous white adipose tissue, TBX1; T-box transcription factor, TFAM; mitochondrial transcription factor A, TMEM26; transmembrane protein 26, UCP1; uncoupling protein 1, ↑; an increase in the experimental group compared to the control group.

**Table 3:** *In vivo* evidence for the increase of non-shivering thermogenesis by flavonoids.

Flavonoids		Subjects	Treatments	Outcomes	Authors
Flavone	Luteolin	Male C57BL/6 mice	HFD with 0.01% luteolin for 12 weeks	↑ O_2_ consumption and CO_2_ production ↑ BAT activity ↑ SWAT browning ↑ AMPK/PGC1α signaling	Zhang et al. [33]
	Olive leaf extract (luteolin)	Male C57BL/6N mice	HFD with 0.15% olive leaf extract for 8 weeks	↓ Body weight and fat pad weight ↑ Browning and mitochondrial biogenesis in EWAT	Shen et al. [43]
	Apigenin mixed with naringenin	Male C57BL/6 mice	Apigenin/naringenin (80 mg/kg) for 2 weeks	↑ UCP1 in BAT	Thaiss et al. [46]
	Sudachitin	C57BL/6 J mice and db/db mice	HFD with 5 mg/kg sudachitin for 12 weeks	↓ Body weight, subcutaneous and visceral fat contents ↑ O_2_ consumption and energy expenditure ↑ *Ucp1* in SWAT	Tsutsumi et al. [50]
Flavonol	Onion peel Extract (quercetin)	Male SD rats	HFD with 0.36 and 0.72% onion peel extract for 8 weeks	↓ Body weight and weights of total visceral, retroperitoneal, and mesenteric fat fads ↑ *Ucp1* and *Cpt1α* in EWAT	Moon et al. [52]
	Onion peel extract (quercetin)	Male C57BL/6 mice	HFD with 0.5% onion peel for 8 weeks	↑ Adipocyte browning in RWAT and SWAT	Lee et al. [54]
	Quercetin	Male C57BL/6 mice	HFD with 0.1% Quercetin for 12 weeks	↓ Body weight and weights of EWAT and SWAT ↑ AMPK phosphorylation, and SIRT1 expression in EWAT ↑ *Ucp1* in BAT	Dong et al. [53]
	Rutin	Male C57BL/6 J mice and C57BLKS/J-(db/db) mice	HFD with rutin (1 mg/mL) in drinking water for 10 weeks	↑ Mitochondria biogenesis and whole-body energy expenditure ↑ BAT activity and SWAT browning	Yuan et al. [57]
	Rutin	Female polycystic ovary syndrome SD-rats	Rutin (100 mg/kg) in drinking water for 3 weeks	↑ UCP1, *Ucp1*, *Pparα*, *Pgc1α*, *Pgc1*β, *and Cpt1*α in BAT, ↑ Body temperature	Hu et al. [60]
	Myricetin	Male C57BLKS/J-(db/db) mice	HFD with myricetin (400 mg/kg) in drinking water for 14 weeks	↓ Body weight, fat mass, and blood glucose ↑ Body temperature and oxygen consumption ↑ BAT activity ↑ IWAT browning and mitochondrial biogenesis	Hu et al. [61]
	*Gelidium elegans* (hesperidin rich)	Male ICR mice	HFD with *Gelidium* elegans extract (50, 200 mg/kg/day) for 7 weeks	↓ Body weight, fat mass, plasma insulin, and TG level ↑ AMPK phosphorylation in BAT and BAT activity	Choi et al. [68]
	G-hesperidin	Male Wistar rats	60 mg of G-hesperidin by acute oral administration	↑ BAT-sympathetic nerve activity ↑ Body temperature ↓ Cutaneous sympathetic nerve activity	Shen et al. [70]
Flavanal	Green tea extract (catechin and EGCG)	Male SD rats	Chow diet with catechin and EGCG (0–200 µM) in drinking water	↑ BAT activity and O_2_ uptake rate	Dulloo et al. [75]
	Green tea (EGCG)	Male SD rats	HFD with green tea extract (20 g/kg)	↓ Body weight, digestibility ↑ Energy expenditure ↑ BAT density and β-adrenoceptor activity	Choo et al. [76]
	Tea catechins (TC)	Male SD rats	Low fat diet (LFD) and HFD with 0.5% TC for 5 weeks	↑ *Ucp1* in BAT of LFD with TC group - No significant difference between HF and HF with TC	Nomura et al. [77]
	Green tea catechins	Male SD rats	LFD and HFD with green tea catechins (100 mg/kg) for 5 weeks	↑ PPARδ, UCP1, *Ucp1,* and CPT1α in WAT and BAT	Yan et al. [78]
	Oolong, black, and pure teas	Male ICR mice	7 days consumption with tea boiled with 2 g tea leaves in 100 mL	↓ Weight of WAT, ↑ AMPK phosphorylation in WAT and BAT, ↑ UCP1 in WAT	Yamashita et al. [82]
	Catechin	Healthy young women	540 mg/day; catechin for 12 weeks	↑ BAT density	Nirengi et al. [84]
	Epigalo catechin gallate (EGCG)	Healthy young men	Cold exposure for 3 h after 1600 mg of EGCG and 600 mg of caffeine intake	↑ Energy expenditure ↓ Shivering thermogenesis	Gosselin et al. [85]
	Cocoa flavanols	Male ICR mice	10 mg/kg cocoa flavonoid fraction or epicatechin.	↑ BAT activity ↑ AMPK phosphorylation in BAT ↑ Plasma catecholamine level	Matsumura et al. [85]
	Cocoa flavanols	Male Wistar rat	HFD with cocoa powder 1 g/kg, cocoa extract 100 mg/kg and (-)-epicatechin 10 mg/kg for 8 weeks	↑ *Ucp1*, *Pparγ*, *Pparα*, *Sirt1,* and *Pgc1α* in BAT ↑ AMPK phosphorylation in BAT ↑ Plasma catecholamine level	Rabadan-Chávez et al. [88]
	Epicatechin	Male Wistar rats	HFD for 5 weeks with (-)-epicatechin (1 mg/kg) for additional 2 weeks	↑ EWAT browning ↓ Body weight	Gutiérrez-Salmeán et al. [89]
Anthocyanin	Bilberry Extract	Male KK-Ay mice	27 g/kg diet for 5weeks	↑ AMPK in SWAT and skeletal muscle	Takikawa et al. [96]
	Cyanidin-3-glucoside (C3G)	Male C57BLKS/J-(db/db) mice	C3G dissolved in drinking water (1 mg/mL) for 16 weeks	↑ Energy expenditure representing oxygen consumption ↑ BAT activity ↑ Body temperature and mitochondrial biogenesis in BAT ↑ SWAT browning ↓ Body weight gain, and weight of EWAT and SWAT	You et al. [98]
Isoflavone	Puerariae flower (PFE) and isoflavone fraction (PF)	Male C57BL/6J mice	HFD with 5% PFE and PF isoflavone fraction for 6 weeks	↑ Energy expenditure representing oxygen consumption ↑ UCP1-positive area in BAT ↓ Body weight gain, and weight of EWAT and SWAT	Kamiya et al. [104]
	Isoflavone mixture	Long-Evans male and female rats	600 µg of phytoestrogens/g of diet	↑ Core body temperature during light cycle ↑ *Ucp1* in BAT	Lephart et al. [105]
	Daidzein	Male Wistar rats	LFD and HFD with 50 mg/kg for 2 weeks	↑ *Ucp1* in BAT in HFD	Crespillo et al. [106]
	Formononetin	Male C57BL/6J mice	HFD with 0.1, 1, and 10 mg of formononetin	↑ SWAT browning ↑ Small and multilocular lipid droplets in SWAT	Gautam et al. [108]

AMPK; 5′ AMP-activated protein kinase, BAT; brown adipose tissue, CPT1α; carnitine palmitoryl transferase 1 alpha, EWAT; epididymal white adipose tissue, **H**FD; high fat diet, IWAT; Inguinal white adipose tissue, LFD; low fat diet, PPARγ; peroxisome proliferator-activated receptor gamma, PPARα; PPAR alpha, PPARδ; PPAR delta, PGC1α; PPARγ coactivator 1 alpha, PGC1*β;* PPARγ coactivator 1-beta, RWAT; retroperitoneal white adipose tissue, SD; Sprague Dawley, SIRT1; silent mating type information regulation 2 homolog 1, SWAT; subcutaneous white adipose tissue, TAG; triacylglycerol, UCP1; uncoupling protein 1, ↑; an increase in the experimental group compared to the control group, ↓; a decrease in the experimental group compared to the control group.

## Abbreviations

ACC	acetyl-coenzyme A carboxylase
ACO	acyl-coenzyme A oxidase
AMPK	AMP-activated protein kinase
AP2	adipocyte protein 2
ATP5α	ATP synthase F1 subunit alpha
BAT	brown adipose tissue
β-AR	beta-adrenergic receptor
BMP7	bone morphogenetic protein 7
C/EBPβ	CCAAT/enhancer-binding protein beta
CD137/ TNFRSF9	tumor necrosis factor receptor superfamily member 9
CIDEA	cell death-inducing DNA fragmentation factor alpha (DFFA)-like effector a
CITED	cbp/p300-interacting transactivator
COMT	catechol-*O*-methyltransferase
CPT1α	carnitine palmitoyltransferase 1 alpha
DHEA	dehydroepiandrosterone
DIO2	deiodinase 2
EG	epicatechin gallate
EGC	epigallocatechin
EGCG	epigallocatechin gallate
ELOVL3	elongation of very-long chain fatty acids-like 3
EWAT	epididymal white adipose tissue
FAS	fatty acid synthase
FGF 21	fibroblast growth factor 21
HFD	high-fat diet
HSL	hormone sensitive lipase
IWAT	inguinal white adipose tissue
LDL	low density lipoprotein
MAPK	mitogen-activated protein kinase
MCAFA	medium-chain acyl-coenzyme A
ME	mulberry extract
Myf5	myogenic factor 5
NHANES	National Health and Nutrition Examination Survey
NRF1	nuclear respiratory factor 1
NDUFB8	246 NADH Dehydrogenase (Ubiquinone) 1 Beta Subcomplex, 8
OLE	olive leaf extract
OPE	onion peel extract
PGC1α	peroxisome proliferator-activated receptor gamma coactivator 1 alpha
PLIN	perilipin
PKA	protein kinase A
PKC	protein kinase C
PPARα	peroxisome proliferator-activated receptor alpha
PPARβ	peroxisome proliferator-activated receptor beta
PPARγ	peroxisome proliferator-activated receptor gamma
PRDM16	positive regulatory domain containing 16
PCOS	polycystic ovary syndrome
RWAT	retroperitoneal white adipose tissue
SWAT	subcutaneous white adipose tissue
SIRT1	silent mating type information regulation 2 homolog 1
SDHB	succinate dehydrogenase complex iron sulfur subunit B
TAG	triacylglycerol
TBX1	T-box transcription factor 1
TFAM	mitochondrial transcription factor A
TMEM 26	transmembrane protein 26
UCP1	uncoupling protein 1
UCP2	uncoupling protein 2
UCP3	uncoupling protein 3
UQCRC2	ubiquinol-cytochrome c reductase core protein 2
VLDL	very-low density lipoprotein
WAT	white adipose tissue
WNT	wingless type
WNT10b	wingless type MMTV integration site family, member 10b
